# Antimicrobial Prophylaxis in Elective Orthopaedic Surgical Procedures at the Tertiary Care Hospital: A Prospective Observational Study

**DOI:** 10.3390/antibiotics14100968

**Published:** 2025-09-25

**Authors:** Milica Kosić, Branislava Miljković, Vedrana Barišić, Sandra Vezmar-Kovačević, Marija Jovanović, Katarina Vučićević, Tijana Kovačević

**Affiliations:** 1 Department of Clinical Pharmacy and Clinical Investigation, Hospital Pharmacy, University Clinical Centre of the Republic of Srpska, 12 beba bb, 78000 Banja Luka, Bosnia and Herzegovina; vedrana.barisic@med.unibl.org (V.B.); tijana.kovacevic@med.unibl.org (T.K.); 2 Department of Pharmacokinetics and Clinical Pharmacy, Faculty of Pharmacy, University of Belgrade, Vojvode Stepe No. 450, 11221 Belgrade, Serbia; branislava.miljkovic@pharmacy.bg.ac.rs (B.M.); sandra.vezmar-kovacevic@pharmacy.bg.ac.rs (S.V.-K.); marija.jovanovic@pharmacy.bg.ac.rs (M.J.); katarina.vucicevic@pharmacy.bg.ac.rs (K.V.); 3 Department of Pharmacy, Faculty of Medicine, University of Banja Luka, Save Mrkalja No. 14, 78000 Banja Luka, Bosnia and Herzegovina

**Keywords:** surgical site infection, antibiotic prophylaxis, orthopaedic surgery, elective surgical procedure, risk factors

## Abstract

**Objective:** Surgical antimicrobial prophylaxis (SAP) is vital for preventing surgical site infections (SSIs). This study evaluated adherence to local SAP guidelines and assessed SSI-related risk factors in elective orthopaedic surgeries. **Methods:** A prospective observational study was conducted at a tertiary care hospital between August and October 2023. Patients were categorised into two groups: those receiving guideline-adherent SAP (SAP group) and those with inadequate SAP (ISAP group), defined by incorrect dosing or prolonged duration. Various patient- and procedure-related SSI risk factors were compared between groups. **Results:** Among 82 patients, 81 received SAP, but only a small proportion received it correctly. Most deviations were due to extended duration and incorrect dosing, resulting in 90.20% non-adherence. Despite this, no significant differences in known SSI risk factors were observed between the two groups, and no SSIs were reported during the study period. **Conclusions:** Non-adherence to SAP guidelines was widespread, mainly due to extended prophylaxis. Importantly, non-adherence was not associated with increased SSI risk, nor was it linked to higher baseline patient risk factors, suggesting that decisions were influenced more by clinical routine than by patient-specific risk. These findings emphasize the need to strengthen staff education and adherence to guidelines, thereby supporting antimicrobial stewardship in resource-limited settings.

## 1. Introduction

Surgical antimicrobial prophylaxis (SAP) is a critical intervention for preventing surgical site infections (SSIs), a major contributor to postoperative morbidity. While international guidelines [1,2,3] and local protocols at the University Clinical Centre of Republic of Srpska (UCC RS) [4] provide explicit recommendations for SAP in orthopaedic surgery, evidence suggests widespread non-compliance across healthcare settings. Inappropriate or excessive antibiotic use can lead to adverse outcomes, including drug side effects, antimicrobial resistance, and escalated treatment costs [1,2,3,4].

While global studies have documented deviations from SAP protocols [5,6], there is a lack of published data on SAP adherence in Bosnia and Herzegovina (BIH), particularly within elective orthopaedic surgery. A review of the available literature reveals a paucity of data on perioperative antibiotic compliance, particularly within low-resource healthcare settings [7] such as BIH. As a healthcare system constrained by limited diagnostics, therapeutic access, staff shortages, and infrastructural deficiencies, BIH encounters significant challenges in ensuring adherence to SAP guidelines. These systemic barriers may contribute to suboptimal SAP compliance. Moreover, no studies to date have systematically evaluated the factors associated with SSIs among patients undergoing surgical procedures in this context, underscoring a critical gap in evidence necessary for informed policy and practice improvements. Addressing this gap is essential to guide local clinical practice, optimize SAP adherence, and ultimately reduce SSI rates in BIH orthopaedic surgery.

This study aimed to evaluate adherence to SAP guidelines in elective orthopaedic procedures at UCC RS, with particular focus on antibiotic dosing, timing, and duration. Additionally, it explores patient- and procedure-specific risk factors associated with SSIs in this setting.

## 2. Results

Of 105 patients initially considered, 23 were excluded due to surgery delays (13), mortality (4), transfers to other institutions (4), or reoperations (2), resulting in a final sample of 82 patients.

### 2.1. Patient and Procedure Characteristics

The median age of cohort was 65 years (IQR: 21.25). More than half of the patients were retired, while workers accounted for most of the remaining group. Smoking and alcohol consumption were frequent, affecting around 40% and 35% of patients, respectively. Only a minority reported allergies, most commonly to NSAIDs or penicillin. Other demographic characteristics are shown in Table 1.

Orthopaedic procedures ranged from minimally invasive interventions to total joint arthroplasties, with almost half of patients (45.10%) undergoing total joint replacement. Implant-related surgeries dominated the cohort, consistent with the demographic profile of older adults (Table 2).

### 2.2. SAP Compliance and Antibiotic Use

Inadequate SAP was identified in 74 of 82 patients (90.20%), who were assigned to the Inadequate SAP (ISAP) group. The remaining 8 patients (9.80%) either received SAP in accordance with guideline recommendations or did not receive it when not indicated; these individuals comprised the SAP group. Among the total cohort, three patients reported a penicillin allergy. One did not receive SAP and was included in the SAP group. The other two received gentamicin—an agent not recommended when beta-lactam cross-reactivity is anticipated—and were therefore assigned to the ISAP group.

Among those who received SAP (N = 81), only 8.60% met both dose and duration criteria, while over three-quarters (77.80%) received SAP for longer than the recommended. Most commonly, patients were given a single preoperative dose followed by repeated 1 g doses every 8 h for 24 h (48.10%), indicating a tendency toward “routine-based” rather than risk-adjusted prescribing. A smaller group received SAP for up to 8 days, though this extended use did not correspond with higher documented risk (Table 3). Despite this overuse, no SSIs were recorded in either the SAP or ISAP groups during the formal two-week follow-up.

The pattern of antibiotic use was consistent: 97.30% of patients in the ISAP group received cefazolin, and only two patients received gentamicin due to allergy, which itself represented a deviation from recommended alternatives. A positive and statistically significant correlation between body weight and cefazolin dose suggests that dose adjustments were at least partially weight-informed (r = 0.433, *p* < 0.001; Table 4), though still frequently above recommended thresholds.

### 2.3. Risk Factors and Group Comparisons

No statistically significant differences were observed between SAP-adherent and non-adherent patients with respect to known SSI risk factors, including age, BMI, smoking, diabetes, or type of procedure (Table 5). This lack of differentiation suggests that antibiotic overuse was not guided by individualized risk but more likely by institutional habit or clinical caution.

Interestingly, patients undergoing low-risk, minimally invasive procedures such as arthroscopy or joint stabilization almost universally received SAP, contrary to recommendations. Similarly, implant placement was not significantly associated with prolonged or excessive SAP (*p* = 0.273), although such procedures are often assumed to justify extended prophylaxis.

## 3. Discussion

Excluding the lack of information regarding the exact timing of antibiotic administration, the key deviation from SAP guidelines was prolonged duration of SAP, observed in 77.80% of patients. Comparison was made with Williams and Gustilo’s [8] (1984) trial of 1.341 arthroplasties, which showed no added benefit of extending cefazolin beyond 24 h. It is encouraging that only 8.86% of patients from our research who received cefazolin for SAP had it administered for more than 24 h, with the longest duration recorded being 8 days. None of them developed SSI, highlighting that prolonged SAP did not correlate with increased risk. Nearly half (48.10%) of patients in our study received a cefazolin prophylaxis regimen, consisting of a single preoperative dose followed by 1 g doses administered at 8 h intervals within a 24 h. This dosing approach demonstrates a considerable level of alignment with the comparative study.

Newer expert consensus, supported by Morrison et al.’s 2012 meta-analysis of 921 patients reported no difference in SSI rates between single and multiple (3–4) doses within 24 h [9]. Despite this, 68.35% of patients in our study who received cefazolin had more than a single dose within 24 h. In our older (65 vs. 41.6 y), comorbidity-heavy cohort, suffering mostly from diabetes mellitus (11% vs. 3.5%), extended prophylaxis was common, likely reflecting cautious practice rather than higher baseline infection risk. Additionally, BIH’s low-resource status must be taken into consideration. These limitations may contribute to a perceived elevated risk of postoperative infections and may partly explain the preference among surgeons for a more conservative, prolonged antibiotic prophylaxis approach, even within the confines of existing guidelines. The healthcare system in BiH faces well-documented challenges, including limited diagnostic resources, inconsistent implementation of protocols, and a lack of antimicrobial stewardship infrastructure. These systemic issues likely contribute to routine-based rather than evidence-based prescribing practices.

The choice of prophylactic agent remains crucial. In our study, cefazolin was the preferred agent and yielded favorable outcomes, reinforcing its role as first-line prophylaxis [3,6,10]. Evidence from other studies confirms that drug choice affects SSI outcomes. For instance, Bratzler et al. (2013) [3] demonstrated higher SSI rates with vancomycin monotherapy compared to cefazolin, particularly in orthopaedic procedures, emphasizing cefazolin’s superior efficacy. Similarly, studies show that substituting clindamycin in beta-lactam–allergic patients is associated with higher SSI risk compared with cefazolin [10,11]. Some patients received prolonged or higher-dose prophylaxis, even though the presence of risk factors was not increased, suggesting that such adjustments likely reflect cautious practice rather than a demonstrable need, highlighting potential areas to optimize antibiotic use and avoid unnecessary prolongation.

When compared with other low-resource settings, adherence in our institution was relatively higher. Argaw et al. (2017) in Ethiopia [12] found that 87.30% of patients received SAP beyond 24 h, versus only 8.90% in our study, despite their patients being younger (mean 35.7 vs. 65 years) and healthier (diabetes prevalence 1.50% vs. 11%). Their SSI rate was 8.30%, likely influenced by incomplete follow-up, compared to zero infections in our official 14-day record review. This contrast suggests that better adherence can be achieved even in constrained systems.

Comparison of patient- and procedure-related SSI risk factors between the ISAP and SAP groups was limited by the scarcity of studies analyzing individual risk factors in detail. Some of the available data, as in one Swiss study [13], indicates a significantly higher number of patients from ISAP group who had an implant placed during surgery compared to a group of patients from SAP group who had an implant (*p* < 0.001) unlike our study, which showed no statistical difference between the two groups (*p* = 0.273). Overall frequency of prolonged SAP in this study was 12% during a 90 days post-operative follow-up. The cohort was younger than ours (65 vs. 58 years) with SAP prolongation associated with older age (63 vs. 58 years, *p* < 0.05), unlike in our cohort where statistical difference in age between groups was not noticed (65.50 vs. 65. years, *p* = 0.756).

In our cohort 90% of patients undergoing minimally invasive orthopaedic procedures, specifically arthroscopy and joint stabilization, received SAP, despite the low-risk profile associated with these interventions. Retrospective study by Qi et al. [14] (2018) reported lower percent of deviations—46.30% of 1.326 arthroscopy patients. The two cohorts were demographically comparable, with both studies reporting a mean patient age of 41 years and similar BMI values (25.11 kg/m^2^ vs. 24.80 kg/m^2^). Our study included one patient with diabetes mellitus (14.3% of the arthroscopy subgroup), compared to approximately 8% in each group reported by Qi et al. Contrary to expectations, neither study found diabetes mellitus to be a significant risk factor for SSI. Despite this, no SSIs were observed. These findings highlight an overutilization of SAP in low-risk patients and support the growing body of literature recommending a more selective, evidence-based approach to prophylaxis in minimally invasive orthopaedic surgery.

Our study demonstrates that, despite frequent deviations from SAP guidelines—particularly regarding duration and dosage—these did not increase the incidence of SSIs within the observed timeframe. The prevalent non-adherence appears to be driven by physician caution, limited awareness of updated recommendations, and systemic constraints inherent to a low-resource healthcare setting. Nevertheless, outcomes in our cohort were better than those reported in similar resource-constrained countries, indicating that adherence and stewardship can be improved even under challenging conditions.

From a clinical perspective, prolonged prophylaxis in our cohort likely reflects unnecessary caution rather than higher patient risk. While no SSIs were observed, extending SAP offers no additional protection and exposes patients to avoidable risks such as antimicrobial resistance [15,16], drug toxicity [16,17,18,19], and increased cost [20]. The absence of SSIs may also be partly explained by the relatively short follow-up period; some prosthetic joint infections manifest months after surgery. Thus, while our results support the safety of short-course SAP, long-term surveillance is needed, particularly for implant-bearing patients.

### Limitations

A primary limitation of this study is the absence of data on the exact timing of SAP administration relative to the initial incision, which hampers a comprehensive assessment of prophylaxis adequacy. Additionally, information on total blood loss, which could justify extended SAP dosing, was unavailable.

Formal follow-up was conducted at 14 days postoperatively due to the limited timeframe of the study. To ensure comprehensive monitoring, all patients’ medical records were retrospectively reviewed at 30, 60, and 90 days revealing no superficial or deep SSIs. While longer prospective follow-up could better capture delayed infections, some of which may manifest months post-procedure, the current data reliably capture early outcomes. A primary limitation of this study is the absence of data on the exact timing of SAP administration relative to the initial incision, which hampers a comprehensive assessment of prophylaxis adequacy. Additionally, information on total blood loss, which could justify extended SAP dosing, was unavailable.

The study intentionally included several pre-specified patient- and procedure-related risk factors (such as age, BMI, diabetes mellitus, smoking, procedure duration, and implant use) to assess their impact on SSI outcomes and prophylactic practices. These factors are therefore not considered confounding variables. Their inclusion allows interpretation of deviations from guideline-based prophylaxis as likely reflecting cautious clinical decision making rather than unaccounted bias.

The relatively small sample size and the focus exclusively on elective procedures performed under controlled conditions. Non-elective interventions, which are inherently more heterogeneous due to variations in urgency, wound contamination, patient comorbidities, age, and other factors, were not included. Consequently, the findings may not be directly generalizable to urgent or emergent surgical settings. Future research could address non-elective procedures with tailored study designs to capture meaningful outcomes in these complex scenarios.

Additionally, cefazolin was the predominant prophylactic agent used in our cohort, and outcomes across other antibiotic classes were not compared, which represents an area for future research.

Despite these limitations, the study provides meaningful insights into SSI rates and the effectiveness of standard prophylactic regimens in the observed population, particularly within a low-resource healthcare setting.

## 4. Materials and Methods

### 4.1. Study Design and Data Collection

A systematic, prospective, observational study was conducted at the Orthopaedic Ward of the UCC RS, Banja Luka, BIH, from 1 August 2023, to 31 October 2023. The study protocol received approval from the institutional Ethics Committee (Ref. No.: 01-19-310-2/23).

Data on patient demographics, surgical characteristics, and SAP administration were retrieved from both written and electronic medical records, prospectively. SSI occurrence was also monitored during the postoperative period.

### 4.2. Participants

The study included patients undergoing elective orthopaedic procedures. Patients with emergency surgeries, elevated inflammatory markers, or receiving antimicrobial therapy upon admission were not included. Data collected included patient initials, age, gender, body mass index (BMI), smoking and alcohol use, allergies, type of surgery, comorbidities, and details of antimicrobial prophylaxis (antibiotic choice, dose, and duration).

Patients were categorised into two groups based to SAP guidelines:

- SAP group: patients managed according to guideline-recommended antibiotic choice, dose, timing, and duration, or not receiving prophylaxis when not indicated.
- ISAP group: patients with any deviation from recommended antibiotic choice, dose, timing, or duration.

### 4.3. SAP Protocol and Adherence Criteria

At the UCC RS, SAP guidelines are based on both local protocols and international recommendations [1,2,3,4,21]. Correct SAP administration was defined by the following criteria:

- Antibiotic choice: First-generation cephalosporins, especially cefazolin, are preferred, with clindamycin or vancomycin reserved for patients with beta-lactam allergy or high MRSA risk [1,2,3,4,21,22,23].
- Dosage: Cefazolin 2 g for patients weighing less than 120 kg and 3 g for those weighing over 120 kg, clindamycin 900 mg and vancomycin 15 mg/kg [24,25,26].
- Timing: Intravenously 30 to 60 min before surgical incision to ensure optimal tissue concentration [24,25,26,27].
- Duration: Single dose, with extension up to 24 h in cases of increased SSI risk, prolonged surgical procedures, or implantation of foreign material. SAP beyond 24 h was considered non-adherent [3,11,22].
- Redosing: Recommended when surgical duration exceeded twice the elimination half-life of the drug (≈3 h for cefazolin) or excessive blood loss (>1500 mL) [11].

### 4.4. Pre-Specified Risk Factors

The study was designed to examine several pre-specified risk factors for SSIs, including patient-related factors such as age, BMI, comorbidities, smoking status, and preoperative hospital stay duration, as well as procedure-related factors such as type of surgery, surgical duration, and implant use. These factors were included to assess their impact on SSI outcomes and on surgeons’ prophylactic practices, such as antibiotic choice, dose, and duration, and were therefore not considered confounding variables.

### 4.5. SSI Definition and Rate

Surgical site infections (SSIs) were defined according to the Centers for Disease Control and Prevention (CDC) criteria [10], including superficial incisional, deep incisional, and organ/space infections. The SSI rate was calculated as the number of patients who developed a surgical site infection divided by the total number of patients in the cohort, expressed as a percentage.

### 4.6. Outcomes

The primary outcome was the number of patients receiving an inadequate dose or duration of antimicrobial prophylaxis. An inadequate dose of cefazolin was defined as any dose other than 2 g for patients weighing under 120 kg or other than 3 g for those weighing over 120 kg. Inadequate duration of prophylaxis was characterized as follows: administration of more than a single dose for elective procedures involving foreign material implantation, or continuation beyond 24 h for procedures involving implanted materials and additional SSI risk factors. Furthermore, the use of SAP in minimally invasive clean procedures without implant placement was deemed inappropriate. This study aimed to assess whether the risk of SSI is increased with a single dose of SAP and to evaluate the justification for the common clinical practice of extending prophylaxis beyond a single dose.

### 4.7. Follow-Up

Formal follow-up of patients was performed at 14 days postoperatively. To further monitor potential SSIs, all patients’ medical records were retrospectively reviewed at 30, 60, and 90 days after surgery.

## 5. Conclusions

This study highlights key opportunities to optimize surgical antimicrobial prophylaxis (SAP) practices within our institution. Deviations from local and international guidelines—particularly regarding prophylaxis duration and its use in minimally invasive procedures—indicate a need to revise current protocols. Notably, there were no differences in SSI risk factors between patients who received adequate versus inadequate prophylaxis, suggesting that deviations were not based on higher baseline risk. Despite these deviations, no SSIs were recorded in the monitored cohort, reinforcing the safety of evidence-based SAP practices even in patients with comorbidities.

These findings emphasize the importance of focusing on staff education regarding current guidelines rather than perceived infection risk. Future directions include implementing educational and audit-based interventions, expanding the study to larger cohorts, and exploring alternative prophylactic strategies in high-risk groups.

Although retrospective review of patient records at 30, 60, and 90 days postoperatively revealed no SSIs, these data were not included in the formal analysis due to the study’s limited duration. We plan to systematically incorporate extended follow-up in future research to provide a more comprehensive evaluation of long-term outcomes and further strengthen institutional SAP protocols.

Ultimately, this study offers real-world insight from a resource-limited setting and may serve as a guide for similar institutions aiming to enhance antibiotic stewardship and patient safety.

## Figures and Tables

**Table 1:** Demographic Characteristics of Patients (N = 82).

Characteristic	Value
**Age (years)** median (IQR *)	65.00 (21.25)
Gender, n (%) of male patients	33 (40.20)
BMI ** (kg/m^2^) median (IQR *)	25.15 (5.60)
Occupation, n (%)	
Retired	42 (51.22)
Worker	34 (41.46)
Student	6 (7.32)
Cigarette Consumption, n (%) of smokers	34 (41.46)
Alcohol Consumption, n (%) of consumers	29 (35.37)
Allergies, n (%)	
NSAIDs ***	2 (2.44)
Paracetamol	2 (2.44)
Sulfamethoxazole/Trimethoprim	2 (2.44)
NSAIDs *** and Chloramphenicol	1 (1.22)
Penicillin and dust mites	1 (1.22)
Penicillin and Ciprofloxacin	1 (1.22)
Penicillin and Sulfamethoxazole/Trimethoprim	1 (1.22)
Pantoprazole	1 (1.22)
Caffetin^®^ (paracetamol, propyphenazone, caffeine, codeine)	1 (1.22)

* IQR—interquartile range; ** BMI—body mass index; *** NSAID—nonsteroidal anti-inflammatory drug.

**Table 2:** Types of Surgery (N = 82).

Characteristic	N (%)
Minimally invasive procedures	
Arthroscopy	7 (8.50)
Joint stabilization	3 (3.70)
Hip surgeries	
Hip osteosynthesis	10 (12.20)
Repair with surgical hardware (e.g., orthopaedic implants, nails, screws, plates, or wires)	
Patellar tendon surgery	12 (14.60)
Osteosynthesis	8 (9.80)
Total joint replacement surgeries	
Total joint arthroplasty	37 (45.10)
Hemiarthroplasty	3 (3.70)
Implant removal	2 (2.40)

**Table 3:** Compliance with recommendations for SAP.

Overall Prophylaxis Use	
Recommendation	N (%)
Prophylaxis indicated according to guidelines	72 (87.80)
Patients who received prophylaxis	81 (98.80)
SAP Adequacy (N = 81 patients who received SAP)	
Adequate antibiotic dose	33 (40.70)
Adequate duration	18 (22.20)
Adequate dose and duration	7 (8.60)
SAP duration with cefazolin (N = 79)	N (%)
Single dose in operating room	18 (22.80)
Single dose + 1 g 8 h later	16 (20.20)
Single dose + 1 g every 8 h, for a total duration of 1 day	38 (48.10)
Single dose + 1 g every 8 h, for a total duration of 3 days	2 (2.50)
Single dose + 1 g every 8 h, for a total duration of 5 days	1 (1.30)
Single dose + 1 g every 8 h up to 8 days	4 (5.10)
Antibiotic Use by Compliance Group	
SAP group (N = 8)	N (%)
Cefazolin	7 (87.5)
No SAP received	1 (12.5)
ISAP group (N = 74)	
Cefazolin	72 (97.30)
Gentamicin	2 (2.70)

**Table 4:** Correlation between body weight and cefazolin dose.

	Mean Value ± Standard Deviation	r	*p*-Value
Body Weight (kg)	78.94 ± 17.168	0.433	<0.001
Cefazolin Dose (grams)	2.54 ± 0.501		

**Table 5:** SSI Risk Factors Between Cohorts.

	ISAP Group (N = 74)	SAP Group (N = 8)	Total (N = 82)	*p*-Value
**Patient related risk factors**				
Age (years), median (IQR *)	65.50 (19.00)	65.00 (24.00)	82 (100.00%)	0.756
Older than 65 years, n (%)	41 (91.10)	4 (8.90)	45 (100.00%)	0.850
BMI in kg/m^2^, median (IQR *)	25.15 (5.80)	22.50 (5.50)	82 (100.00%)	0.064
Smokers, n (%)	32 (97.00)	1 (3.00)	33 (100.00%)	0.308
Diabetes mellitus, n (%)	8 (100.00)	0 (0.00)	8 (100.00%)	0.359
Preoperative hospital stay (days), median (IQR *)	1.00 (2.00)	1.00 (5.00)	82 (100.00%)	0.599
**Procedure related risk factors**				
Type of surgery				
Arthroscopy	7 (100.00)	0 (0.00)	7 (100.00%)	0.344
Joint stabilization	3 (100.00)	0 (0.00)	3 (100.00%)	
Hip osteosynthesis	7 (70.00)	3 (30.00)	10 (100.00%)	
Patellar tendon surgery	11 (91.70)	1 (8.30)	12 (100.00%)	
Osteosynthesis	8 (100.00)	0 (0.00)	8 (100.00%)	
Total joint arthroplasty	34 (91.90)	3 (8.10)	37 (100.00%)	
Hemiarthroplasty	3 (100.00)	0 (0.00)	3 (100.00%)	
Implant removal	2 (100.00)	0 (0.00)	2 (100.00%)	
Duration of the procedure (minutes), median (IQR *)	100 (46.00)	90 (30.00)	82 (100.00%)	0.464
Inserted implant, n (%)	63 (90.00)	7 (10.00)	100 (100.00)	0.273
**Other important procedure characteristics**				
Postoperative drainage use	0	0	0	N/A **
Intraoperative SAP timing data	N/A **	N/A **	N/A **	N/A **
Intraoperative blood loss	N/A **	N/A **	N/A **	N/A **

* IQR—interquartile range; ** N/A—not applicable.

## Data Availability

The data presented in this study are available upon request from the corresponding author due to privacy reasons.

## Abbreviations

The following abbreviations are used in this manuscript:

SAP	Surgical Antimicrobial Prophylaxis
SSI	Surgical Site Infection
UCC RS	University Clinical Centre of Republic of Srpska
MRSA	Methicillin-resistant *Staphylococcus aureus*
BIH	Bosnia and Herzegovina
IQR	Interquartile Range
BMI	Body Mass Index
NSAID	Nonsteroidal Anti-Inflammatory Drug
SAP group	Patients with Adequate SAP
ISAP group	Patients with Inadequate SAP
N/A	Not Applicable

## References

[1] European Centre for Disease Prevention and Control (ECDC) (2012). Surveillance of Surgical Site Infections in European Hospitals—HAI-SSI Protocol, Version 1.02.

[2] Francetić I., Sardelić S., Bukovski-Simonoski S., Santini M., Betica-Radić L., Belina D., Dobrić I., Đapić T., Erdelez L., Gnjidić Ž. (2010). ISKRA Guidelines for Antimicrobial Prophylaxis in Surgery—Croatian National Guidelines. Liječnički Vjesn..

[3] Bratzler D.W., Dellinger E.P., Olsen K.M., Perl T.M., Auwaerter P.G., Bolon M.K., Fish D.N., Napolitano L.M., Sawyer R.G., Slain D. (2013). Clinical practice guidelines for antimicrobial prophylaxis in surgery. Am. J. Health-Syst. Pharm..

[4] Commission for Drugs and Therapy (2016). Prophylactic Use of Antibiotics in Surgery.

[5] Zoutman D., Chau L., McKinnon P.S., Stiver H.G. (1999). Canadian Survey of Prophylactic Antibiotic Use Among Hip-Fracture Patients. Infect. Control Hosp. Epidemiol..

[6] Ierano C., Thursky K.A., Slavin M.A., Marathe A., Ferguson J., Cheng A.C., Thursky K., Worth L.J. (2020). Appropriateness of Orthopaedic Surgical Antimicrobial Prophylaxis Prescribing in Australia: Meaningful Metrics for Surgeons. Infect. Control Hosp. Epidemiol..

[7] Patel V., Callaghan M., Kyaw M.H. (2022). Perioperative care pathways in low- and lower-middle-income countries: Systematic review and narrative synthesis. World J. Surg..

[8] Williams D.N., Gustilo R.B. (1984). The Use of Preventive Antibiotics in Orthopaedic Surgery. Clin. Orthop. Relat. Res..

[9] Morrison S., White N., Asadollahi S., Lade J. (2012). Single versus multiple doses of antibiotic prophylaxis in limb fracture surgery. ANZ J. Surg..

[10] Berríos-Torres S.I., Umscheid C.A., Bratzler D.W., Leas B., Stone E.C., Kelz R.R., Reinke C.E., Morgan S., Solomkin J.S., Mazuski J.E. (2017). Centers for Disease Control and Prevention Guideline for the Prevention of Surgical Site Infection, 2017. JAMA Surg..

[11] Anderson D.J., Podgorny K., Berríos-Torres S.I., Bratzler D.W., Dellinger E.P., Greene L., Nyquist A.C., Saiman L., Yokoe D.S., Maragakis L.L. (2014). Strategies to Prevent Surgical Site Infections in Acute Care Hospitals: 2014 Update. Infect. Control Hosp. Epidemiol..

[12] Argaw N.A., Shumbash K.Z., Asfaw A.A., Hawaze S. (2017). Assessment of surgical antimicrobial prophylaxis in Orthopaedics and Traumatology Surgical Unit of a Tertiary Care Teaching Hospital in Addis Ababa. BMC Res. Notes.

[13] Rohrer F., Maurer A., Noetzli H., Gahl B., Limacher A., Hermann T., Bruegger J. (2021). Prolonged antibiotic prophylaxis use in elective orthopaedic surgery—A cross-sectional analysis. BMC Musculoskelet. Disord..

[14] Qi Y., Yang X., Pan Z., Wang H., Chen L. (2017). Value of antibiotic prophylaxis in routine knee arthroscopy. Der Orthopäde.

[15] Branch-Elliman W., O’Brien W., Strymish J., Itani K.M.F., Chopra T., Schweizer M.L. (2019). Association of Duration and Type of Surgical Prophylaxis with Antimicrobial-Associated Adverse Events. JAMA Surg..

[16] Bartlett J.G. (2002). Antibiotic-Associated Diarrhea. N. Engl. J. Med..

[17] Centers for Disease Control and Prevention (CDC) (2019). Antibiotic Resistance Threats in the United States, 2019.

[18] Stevens V., Dumyati G., Fine L., Fisher S., van Wijngaarden E., Guh A., Salmon D., McDonald L.C. (2011). Cumulative Antibiotic Exposures Over Time and the Risk of *Clostridium difficile* Infection. Clin. Infect. Dis..

[19] Perazella M.A., Markowitz G.S. (2010). Drug-Induced Acute Interstitial Nephritis. Nat. Rev. Nephrol..

[20] Sipahi O.R. (2008). Economics of Antibiotic Resistance. Expert Rev. Anti Infect. Ther..

[21] Australian Government, Australian Institute of Health and Welfare (2023). Elective Surgery. https://www.aihw.gov.au/reports-data/myhospitals/sectors/elective-surgery.

[22] Mangram A.J., Horan T.C., Pearson M.L., Silver L.C., Jarvis W.R. (1999). Guideline for Prevention of Surgical Site Infection. Infect. Control Hosp. Epidemiol..

[23] Whittlesea C., Hodson K. (2019). Clinical Pharmacy and Therapeutics.

[24] Surahio A.R., Khan A.A., Farooq M.U., Fatima I.F. (2010). Single Versus Three-Dose Antibiotic Prophylaxis in Clean and Clean-Contaminated Operations. J. Ayub Med. Coll. Abbottabad.

[25] De Jonge S.W., Gans S.L., Kuijper E.J., Raijmakers P.G., van den Broek P.J., Keus F., Wiezer M.J., Wille J.C., van der Tweel I., van der Lei J. (2017). Timing of Preoperative Antibiotic Prophylaxis in 54,552 Patients and the Risk of Surgical Site Infection: A Systematic Review and Meta-analysis. Medicine.

[26] Steinberg J.P., Braun B.I., Hellinger W.C., Kurz A., Hohmann S.F., Burke J.P., TRAPE Study Group (2009). Timing of Antimicrobial Prophylaxis and the Risk of Surgical Site Infections: Results From the Trial to Reduce Antimicrobial Prophylaxis Errors. Ann. Surg..

[27] Stefánsdóttir A., Robertsson O., Sundberg M., Knutson K., Lidgren L. (2009). Inadequate Timing of Prophylactic Antibiotics in Orthopaedic Surgery: We Can Do Better. Acta Orthop..

